# Rapid prediction of acute thrombosis via nanoengineered immunosensors with unsupervised clustering for multiple circulating biomarkers

**DOI:** 10.1126/sciadv.adq6778

**Published:** 2024-12-11

**Authors:** Kaidong Wang, Shaolei Wang, Samuel Margolis, Jae Min Cho, Enbo Zhu, Alexander Dupuy, Junyi Yin, Seul-Ki Park, Clara E. Magyar, Oladunni B. Adeyiga, Kristin Schwab Jensen, John A. Belperio, Freda Passam, Peng Zhao, Tzung K. Hsiai

**Affiliations:** ^1^ Division of Cardiology, Department of Medicine, David Geffen School of Medicine, University of California Los Angeles, Los Angeles, CA 90095, USA.; ^2^ Department of Bioengineering, Henry Samueli School of Engineering and Applied Science, University of California Los Angeles, Los Angeles, CA 90095, USA.; ^3^ Division of Cardiology, Department of Medicine, Greater Los Angeles VA Healthcare System, Los Angeles, CA 90073, USA.; ^4^ Department of Haematology, Royal Prince Alfred Hospital, Sydney, New South Wales 2050, Australia.; ^5^ Central Clinical School, Faculty Medicine and Health, The University of Sydney, Sydney, New South Wales 2006, Australia.; ^6^ Department of Pathology and Laboratory Medicine, David Geffen School of Medicine, University of California Los Angeles, Los Angeles, CA 90095, USA.; ^7^ Division of Infectious Diseases, Department of Medicine, David Geffen School of Medicine, University of California Los Angeles, Los Angeles, CA 90095, USA.; ^8^ Division of Pulmonary and Critical Care Medicine, David Geffen School of Medicine, University of California Los Angeles, Los Angeles, CA 90095, USA.

## Abstract

The recent SARS-CoV-2 pandemic underscores the need for rapid and accurate prediction of clinical thrombotic events. Here, we developed nanoengineered multichannel immunosensors for rapid detection of circulating biomarkers associated with thrombosis, including C-reactive protein (CRP), calprotectin, soluble platelet selectin (sP-selectin), and D-dimer. We fabricated the immunosensors using fiber laser engraving of carbon nanotubes and CO_2_ laser cutting of microfluidic channels, along with the electrochemical deposition of gold nanoparticles to conjugate with biomarker-specific aptamers and antibody. Using unsupervised clustering based on four biomarker concentrations, we predicted thrombotic events in 49 of 53 patients. The four-biomarker combination yielded an area under the receiver operating characteristic curve (AUC) of 0.95, demonstrating high sensitivity and specificity for acute thrombosis prediction compared to the AUC values for individual biomarkers: CRP (0.773), calprotectin (0.711), sP-selectin (0.683), and D-dimer (0.739). Thus, a nanoengineered multichannel platform with unsupervised clustering provides accurate and efficient methods for predicting thrombosis, guiding personalized medicine.

## Teaser

A nanoengineered immunosensor uses machine learning to detect multiple biomarkers for rapid prediction of acute thrombosis.

## INTRODUCTION

Acute viral infections are emerging as increasingly complex and prevalent threats to public health worldwide (*1*–*4*). While the incidence of the illness known as COVID-19 is declining, a growing association has been reported between respiratory viral infections and clinical thrombotic events. A prothrombotic state predisposes patients to acute coronary syndromes, cerebrovascular accidents, and pulmonary embolism (*5*–*8*). The presence of COVID-19–associated thromboses raises concern for worse patient recovery rates (*3*, *5*, *9*–*11*). Reports of these thrombotic events underscore the need for an accurate and rapid prediction tool that can be used to determine appropriate anticoagulation prophylaxis and to prepare for the next wave of the severe acute respiratory syndrome coronavirus 2 (SARS-CoV-2) pandemic (*5*, *12*).

In response to the COVID-19–associated thrombosis crisis, the International COVID-19 Thrombosis Biomarkers Colloquium formulated a panel of recommended biomarkers that can be used to predict the risk of developing thrombosis associated with COVID-19 (*5*). This formulated panel is based on retrospective studies of several thousand patients with COVID-19, which have revealed the presence of elevated blood levels of C-reactive protein (CRP), calprotectin, soluble platelet selectin (sP-selectin), and D-dimer in these patients. However, relying on a single biomarker often falls short in terms of sensitivity and specificity for predicting thrombotic events. Furthermore, a primary obstacle in predicting thrombosis is the complex interplay of multiple variables, such as viral strain, virulence, individual health conditions, genetic predisposition, and social determinants of health (*13*, *14*). A comprehensive analytical approach that uses multiple biomarkers simultaneously may improve the accuracy of predicting thrombotic risk during acute illnesses.

In this context, we have developed nanoengineered multichannel immunosensors for the simultaneous detection of four key biomarkers, using unsupervised clustering to analyze variations in biomarker concentrations. This approach enhances thrombosis prediction by capturing the nuanced interplay of these biomarkers (*15*, *16*). In multichannel detection, the signal-to-noise ratio of the electrodes is determined by the specific binding affinity of aptamers or antibodies to the biomarkers (*17*, *18*). Aptamers and antibodies are immobilized on electrochemically deposited gold nanoparticles (Au NPs) based on fiber laser–engraved carbon nanotube (CNT) electrodes, enabling sensitive and specific detection across multiple biomarkers, in contrast to traditional single-biomarker detection methods like the enzyme-linked immunosorbent assay (ELISA) method that require longer detection times and process optimizations (*19*, *20*). Ultimately, our customized and scalable approach is poised to improve the health care team responses to thrombotic events in future pandemics.

Following biomarker detection and quantification, we applied a machine learning technique called unsupervised clustering to identify inherent patterns within the unlabeled data (*16*, *21*). This approach allowed us to organize the data points into distinct clusters, and the unsupervised clustering algorithms effectively stratified patients based on their thrombotic risk from various illnesses. This stratification is grounded in objective and quantifiable biomarker data, providing a robust framework for the personalized assessment of risk profiles to prevent thrombotic complications, including stroke, pulmonary embolism, and acute coronary syndrome, in high-risk patients (*22*, *23*).

## RESULTS

### Nanoengineered immunosensors with unsupervised clustering to enhance thrombosis prediction

**Fig. 1.**
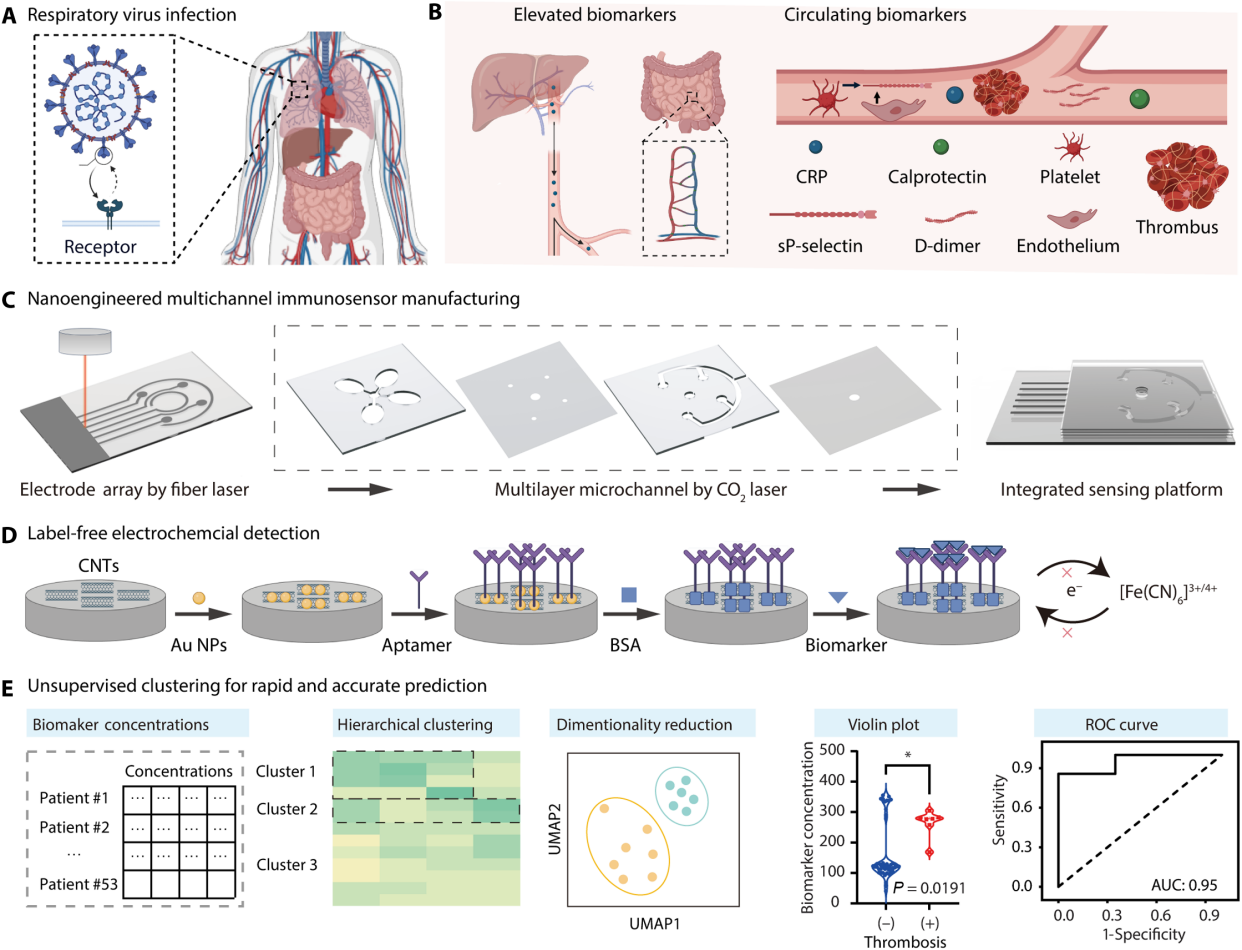
Nanoengineered immunosensors detected multiple biomarkers and used unsupervised clustering for rapid and accurate prediction of acute blood clots. (**A**) Respiratory viruses (e.g., SARS-CoV-2) infected human lung epithelial cells and bound to a specific receptor (e.g., ACE2). (**B**) Invasion by the respiratory virus led to abnormal fluctuations in the concentrations of specific biomarkers in blood circulation due to inflammatory and thrombotic processes, including CRP, calprotectin, sP-selectin, and D-dimer. (**C**) Fabrication of a nanoengineered multichannel immunosensor using fiber and CO_2_ laser technologies. (**D**) Conjugation of aptamers and antibody to the CNT electrodes enabled the rapid electrochemical detection of targeted biomarkers [bovine serum albumin (BSA)]. (**E**) Unsupervised clustering analysis stratified 53 patients into distinct levels of thrombotic risk, using the concentrations of four distinct biomarkers as clustering features. The clustering results were validated with the ICD-10 diagnostic code for thrombosis provided by the UCLA Biobank (PBBR) Core.

Here, we demonstrate a rapid strategy to detect circulating biomarkers in human plasma (Fig. 1). We sought to combine the abnormal fluctuations in the concentrations of four biomarkers associated with thrombosis in COVID-19: CRP, calprotectin, sP-selectin, and D-dimer (Fig. 1, A and B). These biomarkers were measured in deidentified blood samples collected from patients with COVID-19 admitted to the medical center. We fabricated nanoengineered multichannel immunosensors using fiber and CO_2_ laser technologies (Fig. 1C). We conjugated the electrochemically deposited CNTs on the electrodes with aptamers and antibody specific to the biomarkers (Fig. 1D). We detected the concentration of these biomarkers as features of unsupervised clustering to predict the risk for acute blood clots. Subsequently, we compared different unsupervised clustering methods for classifying thrombotic risk from the blood specimens (Fig. 1E). We validated our predicted results against the International Classification of Diseases, Tenth Revision (ICD-10) code for thrombosis provided by the UCLA (University of California, Los Angeles) Pathology Biobanks and Biospecimen Research (PBBR) Core.

To fabricate the nanoengineered multichannel immunosensor, a polyvinyl chloride (PVC) substrate was converted into an electrically conductive substrate (Fig. 1C) by spraying with acid-treated CNTs. Then, a fiber laser (1064 nm, 50W) was used to pattern a predesigned mask onto the CNT substrate to form the multiplexed electrodes. The multichannel detection and wash chambers were engraved with a CO_2_ laser (10,600 nm, 50W) (*19*, *24*, *25*). To achieve electrochemical detection of the thrombosis biomarkers (Fig. 1D), the CNT electrodes were electrochemically deposited with Au NPs and conjugated with aptamers against CRP, sP-selectin, and D-dimer via an Au─S bond (*26*–*28*). The antibody against calprotectin was conjugated to the acid-treated CNT electrode using *N*-(3-dimethylaminopropyl)-*N*′-ethylcarbodiimide (EDC)/*N*-hydroxysuccinimide (NHS)–based chemistry (*29*, *30*). The recognition components of aptamers and antibody on the conductive surfaces changed the charge-transfer resistance (*R*_ct_) at the electrode interface, enabling electrical impedance techniques to be highly effective in assessing the degree of binding affinity (*29*, *30*).

To develop the thrombosis prediction model using the acquired data, we used patient blood specimens (*m*) and biomarkers (*n*) to construct an *m* × *n* computing matrix (Fig. 1E). We used dimensionality reduction and unsupervised clustering algorithms to facilitate the rapid and accurate prediction of thrombotic risk (*21*, *31*). In this representation, each dot corresponds to an individual patient. The concentration of the biomarkers determines the positioning of each dot on the two-dimensional plot. The spatial distance between these plotted points forms the basis of the clustering analysis, which, in turn, dictates the final risk classifications assigned to each patient specimen. This classification enables a visually intuitive and quantitatively robust method to assess the thrombotic risk profile of patients based on the concentration of specific biomarkers. We validated it by comparing it with the ICD-10 diagnostic codes for thrombosis, as provided by the UCLA PBBR Core (*5*, *32*, *33*).

### Rapid detection of biomarkers using nanoengineered multichannel immunosensors

**Fig. 2.**
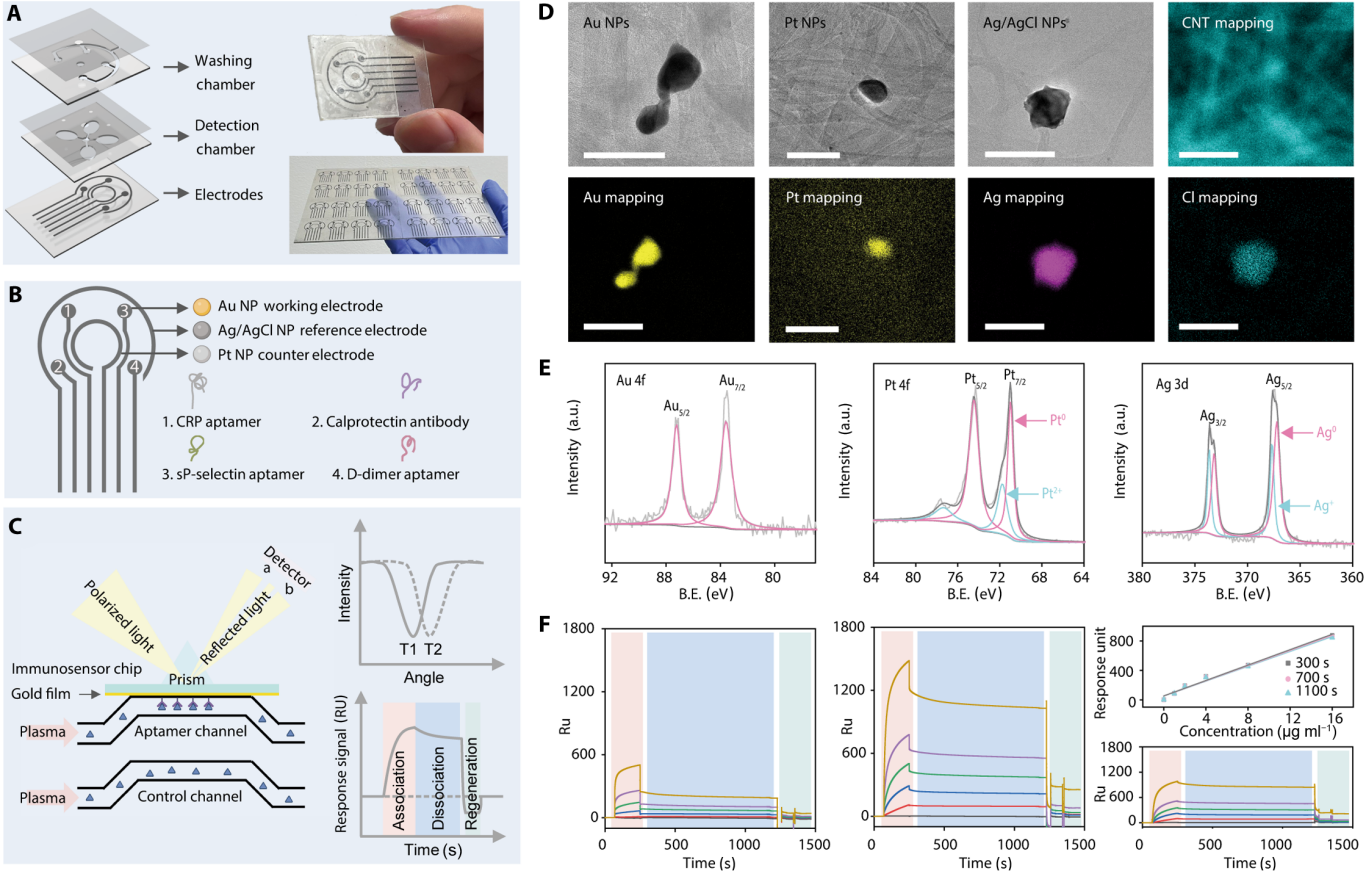
The fabrication and characterization of nanoengineered multichannel immunosensors were demonstrated. (**A**) Schematic representation of a multichannel and multilaminated (10 layers) immunosensor. (**B**) Modification of the CNT electrodes with Au NPs, Ag/AgCl NPs, and Pt NPs, followed by the conjugation of aptamers and antibody. (**C**) Utilization of SPR techniques to study the association, dissociation, and regeneration of targeted biomarkers on the aptamer channel. (**D**) TEM images and EDX elemental mapping of the Au NP working electrode, the Ag/AgCl NP reference electrode, and the Pt NP counter electrode. Scale bars, 100 nm. (**E**) X-ray photoelectron spectroscopy (XPS) spectra of the modified electrodes. (**F**) Comparative responses of control and aptamer channels to targeted biomarkers using SPR techniques. A linear relationship between different dissociation times, response units, and biomarker concentrations was established on the basis of the calibrated response units (RU) via the control and aptamer channels. a.u., arbitrary units; B.E., binding energy.

Given the nature of thrombotic onset in acute infections, rapid detection of a combination of four biomarkers is clinically important. We detected four biomarkers within 1 hour by using a electrochemical immunosensor, using a 5 mM [Fe(CN)_6_]^3+/4+^ solution to capture the distinct impedance signals among various concentration levels of biomarkers. The CO_2_ laser–engraved washing chamber was designed to eliminate residual elements from plasma samples. We verified the regeneration potential of our immunosensor using the surface plasmon resonance (SPR) technique (Fig. 2, C and F), whereby a 0.1 M glycine-HCl buffer (pH 2) removed adherent proteins and effectively regenerated the immunosensor (*34*). To augment the detection range, we used CNTs as the foundational electrodes for their large and specific surface area, enabling conjugation with the aptamers onto the electrochemically deposited Au NPs (Fig. 2, B and D) (*35*). We applied high-resolution transmission electron microscopy (TEM) to image the working electrodes (Au NPs), reference electrodes (Ag/AgCl NPs), and counter electrodes (Pt NPs) (Fig. 2D). Energy-dispersive x-ray (EDX) elemental mapping confirmed the effective integration of Au, Pt, Ag, and Cl elements (Fig. 2D).

We further used x-ray photoelectron spectroscopy (XPS) to elucidate the valence states of the fabricated electrodes (Fig. 2E) (*35*). The Au 4f spectrum revealed a singular peak pair (Au 4f_5/2_/4f_7/2_) at 83.6 and 87.2 eV, indicating the Au^0^ state. In the Pt 4f spectrum, two peak pairs were observed (Pt 4f_5/2_/4f_7/2_) at 74.5 and 71.0 eV and 77.4 and 71.8 eV, corresponding to the Pt^0^ and Pt^2+^ states, indicating strong binding of Pt NPs to the supporting CNT electrode. The Ag 3d spectrum exhibited two peak pairs (Ag 3d_3/2_/3d_5/2_) at 373.2 and 367.2 eV and 367.7 and 373.6 eV, indicating the presence of Ag^0^ and Ag^+^ states and the chlorination of the Ag NPs. Complementing these findings, EDX elemental mapping verified the uniform distribution of Cl elements in the Ag NPs, supporting the efficient synthesis of the Ag/AgCl NP reference electrodes. SPR techniques were used to investigate the association and dissociation kinetics between the modified aptamer immobilized on an Au surface and its targeted biomarker (Fig. 2, C and F) (*36*). The results demonstrate robust interactions between the aptamer and the biomarker, as depicted in Fig. 2F. As the biomarker concentration increased, there was a linear rise in the response signals. This linearity persisted with minimal variation during the dissociation phase, affirming the efficacy of this aptamer-based immunosensor in targeted biomarker detection. The robust interactions were attenuated in a pH 2 buffer solution when rejuvenating the immunosensor surface, suggesting the presence of strong noncovalent forces, including hydrogen bonding and Van der Waals interactions (*37*).

### Performances of nanoengineered multichannel immunosensors in biomarker detection

**Fig. 3.**
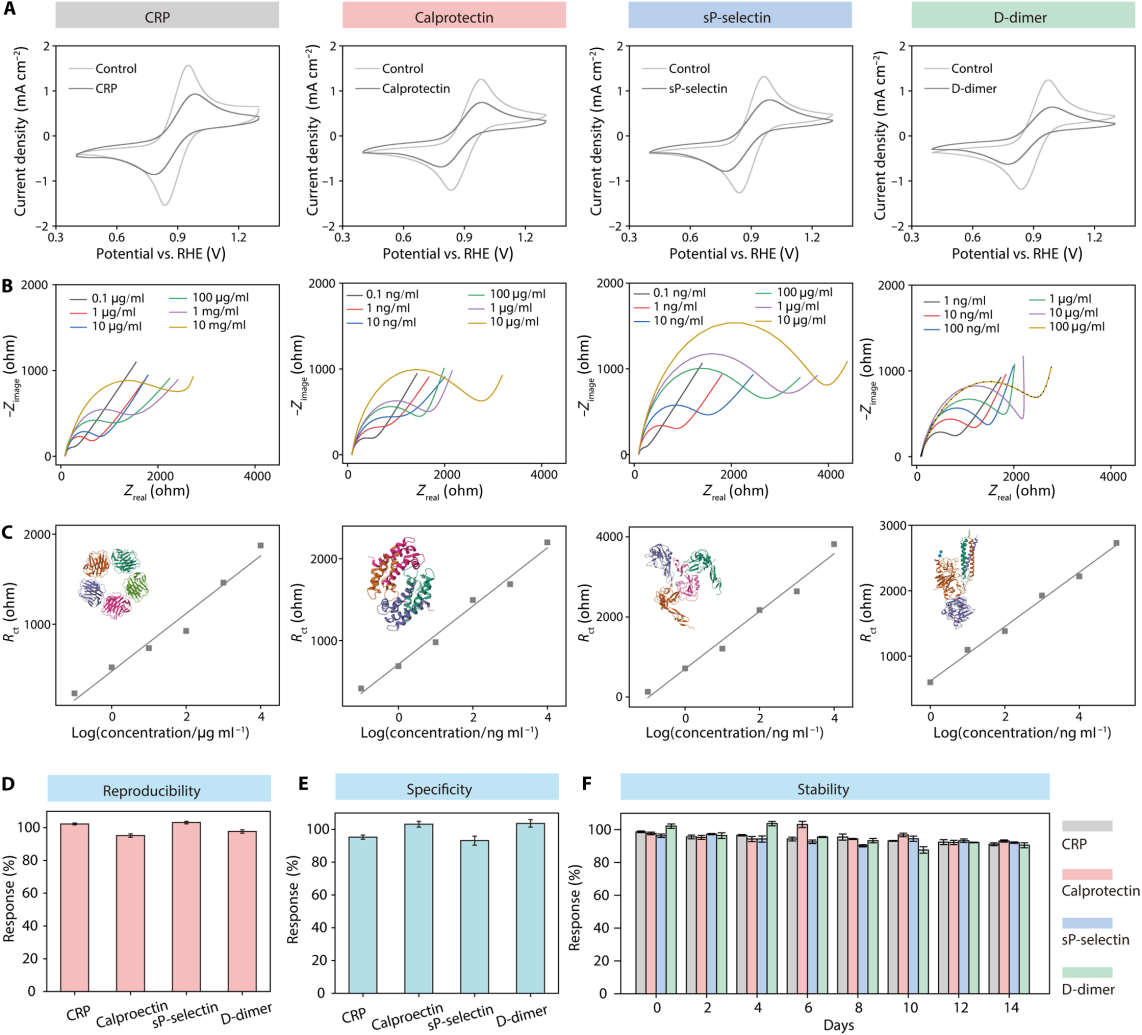
The performance of the nanoengineered multichannel immunosensor was evaluated. (**A**) CV testing compared current density with (gray curve) and without (light gray curve) the targeted biomarkers. (**B**) Electrochemical impedance measurements of the targeted biomarker at various concentrations. (**C**) Linear relationship between charge-transfer resistance and four biomarker concentrations. (**D**) Independent measurements demonstrating the reproducibility of the individual immunosensor (*n* = 6). (**E**) Specificity toward targeted biomarkers in the presence of interferents (*n* = 3). (**F**) Storage stability was established over 14 days. Error bars represent the SD from repeated measurements (*n* = 3) unless otherwise specified.

Cyclic voltammetry (CV) was performed to investigate the electrochemical behavior of the immunosensor before and after the specific adsorption of the targeted biomarker (Fig. 3A). Notably, after the adsorption of the biomarker onto the immunosensor, a pronounced reduction in peak current density was observed. This suggests that the formation of the specific immune complex served as a blocking layer, inhibiting charge transfer on the surface of the electrode. To validate the capability of the immunosensor to detect various concentrations of four biomarkers, we conducted electrochemical impedance spectroscopy (EIS) analysis to examine the linear relationship between multiple concentrations of the targeted biomarkers and charge-transfer resistance (*R*_ct_) using the Randles equivalent circuit model (Fig. 3, B and C) (*38*). In the Nyquist plots (Fig. 3B), the semicircular region at high frequencies correlates with the charge-transfer limited process, while the linear segment at low frequencies is associated with the diffusion-limited process (*38*). A consistent increase in *R*_ct_ values was observed with increasing concentrations of targeted biomarkers. This specific adsorption reduced the charge transfer between the electrodes and the [Fe(CN)_6_]^3+/4+^ redox probe, consistent with our CV findings. As presented in Fig. 3C, the calibration curve illustrates the proportional changes in *R*_ct_ values with the concentrations of targeted biomarkers, indicating robust linear relations within specified ranges. These linear relations were observed over the ranges of 0.1 to 10^4^ μg ml^−1^ for CRP (*R*^2^ = 0.983), 0.1 to 10^4^ ng ml^−1^ for calprotectin (*R*^2^ = 0.987), 0.1 to 10^4^ ng ml^−1^ for sP-selectin (*R*^2^ = 0.982), and 1 to 10^5^ ng ml^−1^ for D-dimer (*R*^2^ = 0.994). The established limits of detection (LODs; S/N = 3) were determined to be 0.023 μg ml^−1^ for CRP, 0.035 ng ml^−1^ for calprotectin, 0.019 ng ml^−1^ for sP-selectin, and 0.035 ng ml^−1^ for D-dimer, as detailed in tables S1 to S4. We first proposed the four-biomarker combination for acute thrombosis prediction and manufactured a four-channel immunosensor to detect the four biomarkers simultaneously. This robust detection capability can be attributed to the large surface area of the CNT-based immunosensor and the high specificity of aptamers and antibody (*35*).

We evaluated the reproducibility, specificity, and storage stability of the manufactured immunosensor for detecting standard and known biomarkers in phosphate-buffered saline (PBS) (pH 7.4) solution. The reproducibility of the nanoengineered multichannel immunosensor was assessed using six independently constructed sensors (Fig. 3D), with relative SD values ranging from 3.2 to 5.8%. To assess specificity, biomarkers were mixed, resulting in distinct sensing signals for each biomarker: 95.3% for CRP, 103.2% for calprotectin, 93.2% for sP-selectin, and 103.7% for D-dimer (Fig. 3E). Specificity measurements were repeated three times. In our evaluation of the immunosensor’s reproducibility (*n* = 6) and specificity (*n* = 3), the repeated measurements showed slight variations, confirming the excellent reproducibility and specificity of our nanoengineered multichannel immunosensor. Next, the long-term storage stability of the nanoengineered multichannel immunosensor was assessed, revealing sustained sensing signals above 91.1% for CRP, 93.1% for calprotectin, 92.1% for sP-selectin, and 90.5% for D-dimer over 2 weeks (Fig. 3F), demonstrating the robust stability of the fabricated immunosensors. In summary, the nanoengineered multichannel immunosensor boasted wide-ranging and rapid detection of multiple circulating biomarkers within 1 hour, compared with ELISA, which takes about 5 hours.

### Unsupervised clustering to enhance thrombosis prediction

**Fig. 4.**
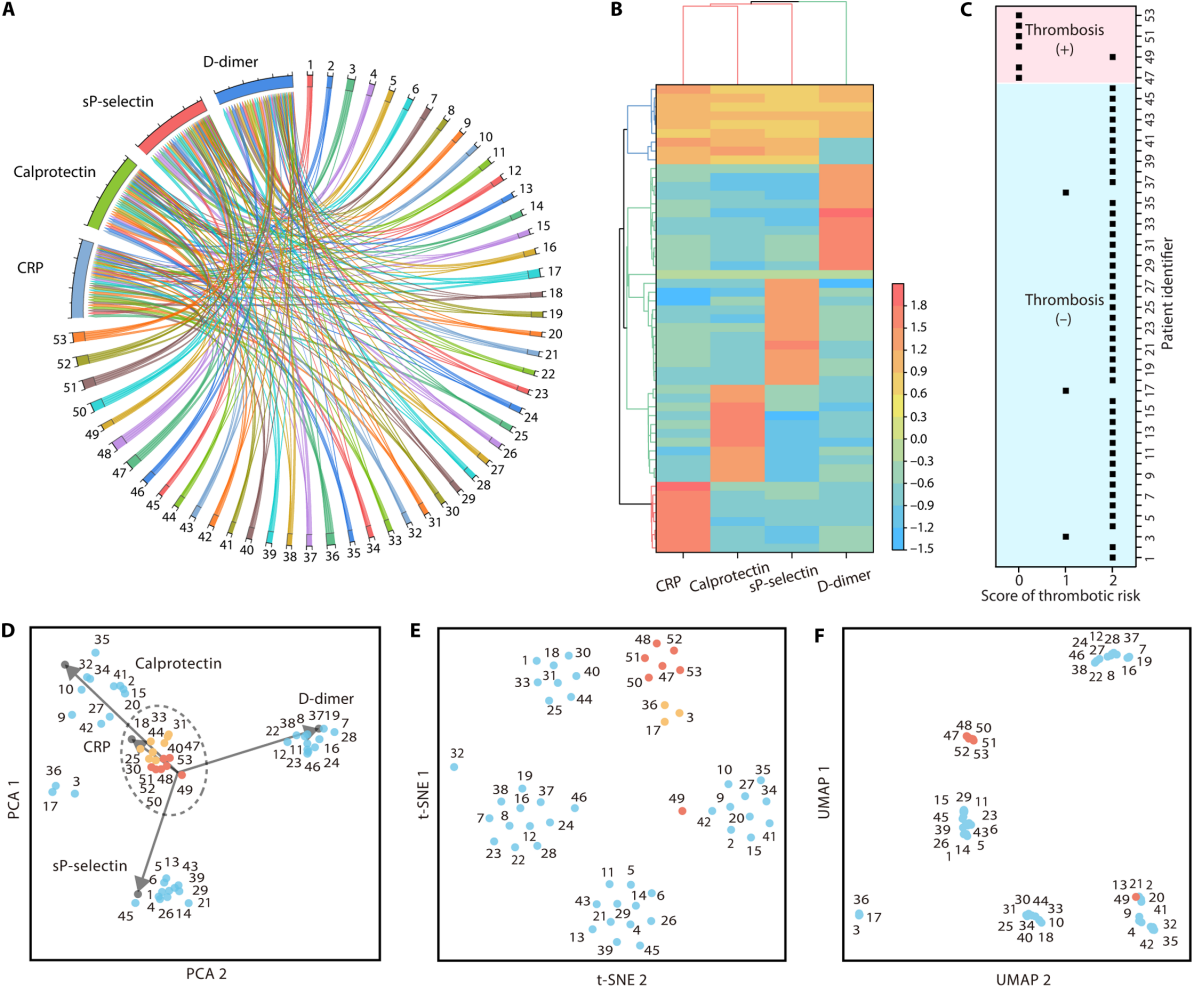
Unsupervised clustering was used for thrombosis prediction with 53 blood specimens from patients with COVID-19. (**A**) A chord diagram illustrates the network relationship among the four biomarkers and 53 patients. (**B**) Hierarchical clustering analysis used the concentrations of the four biomarkers to assess thrombotic risk in the 53 blood specimens. (**C**) Hierarchical clustering outcomes were validated using the ICD-10 diagnostic code for thrombosis. (**D**) PCA analysis was used to cluster the 53 blood specimens. Each arrow represents the influence of a different biomarker concentration in the PCA space. (**E**) t-SNE analysis converted similarities between data points into joint probabilities, facilitating the visualization of patient clusters. (**F**) UMAP analysis preserved both local and global structures, enabling the effective visualization of specimen clusters that are positive or negative for thrombosis.

We used a chord diagram to illustrate the relational network among four biomarkers and 53 blood specimens (Fig. 4A) (*39*, *40*). Notably, the diminished arc span of patient 49 indicates prediction challenges despite the combination of multiple biomarkers. Using the various concentrations detected from the biomarkers, we performed unsupervised hierarchical clustering to categorize blood specimens into three groups, as depicted in Fig. 4B. To ensure patient confidentiality, we adopted a numeric pseudonymization approach for each patient, aligning the clustering outcomes with the ICD-10 diagnostic codes for thrombosis. Notably, the clustering was solely influenced by the concentrations of the four biomarkers. Patient IDs were organized on the basis of diagnostic outcomes to ensure clarity and facilitate comparative analysis.

As illustrated in Fig. 4 (B and C), patients 47, 48, 50, 51, 52, and 53 were identified with the highest thrombotic risk (scores = 2), while patients 3, 17, and 36 exhibited a moderate risk (scores = 1). The other patients were identified with the lowest thrombotic risk (scores = 0). To validate these findings, we cross-referenced them with the ICD-10 codes for thrombosis from hospitalized patients with COVID-19. We reorganized the patient identifiers based on the diagnostic outcomes of positive and negative thrombosis. According to the diagnostic results, 46 patient specimens were negative for thrombosis, and 7 were positive. For comparative analysis, we arranged the negative thrombosis samples from 1 to 46 and the positive thrombosis samples from 47 to 53. We validated the results of the unsupervised hierarchical clustering against the medical ICD code for thrombosis (Fig. 4C) and accurately predicted positive or negative thrombosis in 49 of 53 specimens. A moderate risk rating (score = 1) was assigned to patients 3, 17, and 36, which led to false-positive predictions. In contrast, the lowest risk rating (score = 0) was assigned to patient 49, resulting in a false-negative prediction.

**Fig. 5.**
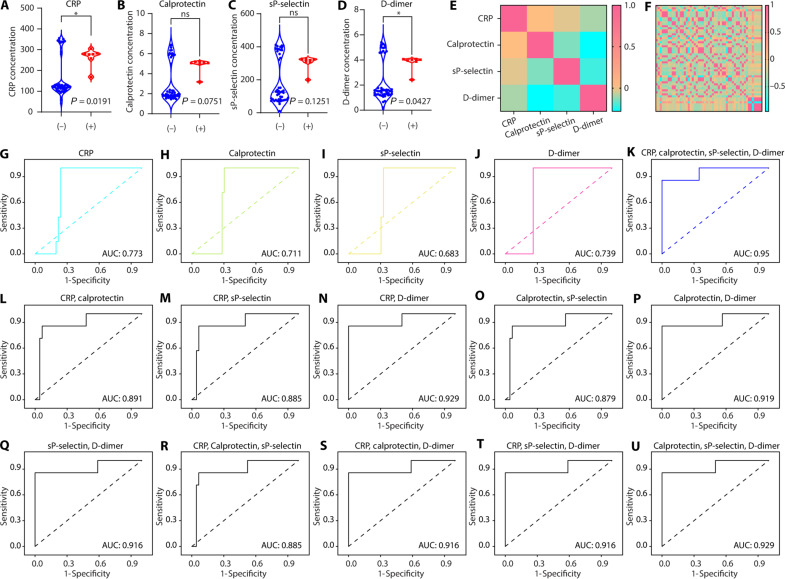
Statistical analysis of the concentrations of four biomarkers was performed on 53 patient blood specimens. (**A** to **D**) Violin plots compare the concentrations of four biomarkers (A: CRP; B: calprotectin; C: sP-selectin; D: D-dimer) between thrombosis-negative (−) and thrombosis-positive (+) patients. (**E**) Proximity matrix among the four biomarkers. (**F**) Proximity matrix among the 53 patients. (**G** to **J**) Receiver operating characteristic (ROC) curve analysis illustrates the relationship between the concentration of a single biomarker (G: CRP; H: calprotectin; I: sP-selectin; J: D-dimer) and thrombosis prediction, using the ICD-10 code for validation. (**K**) ROC curve analysis using the combination of four biomarkers (CRP, calprotectin, sP-selectin, and D-dimer) for thrombosis prediction. (**L** to **Q**) ROC curve analysis using the combinations of two biomarkers for thrombosis prediction (L: CRP and calprotectin; M: CRP and sP-selectin; N: CRP and D-dimer; O: calprotectin and sP-selectin; P: calprotectin and D-dimer; Q: sP-selectin and D-dimer). (**R** to **U**) ROC curve analysis using the combinations of three biomarkers for thrombosis prediction (R: CRP, calprotectin, and sP-selectin; S: CRP, calprotectin, and D-dimer; T: CRP, sP-selectin, and D-dimer; U: calprotectin, sP-selectin, and D-dimer). ns, not significant.

Principal components analysis (PCA), t-distributed stochastic neighbor embedding (t-SNE), and Uniform Manifold Approximation and Projection (UMAP) are dimensionality reduction techniques, each with distinct characteristics suitable for unsupervised clustering (*16*, *41*, *42*). Although the PCA algorithm correctly clustered patient 49 into the thrombosis-positive group, many false-positive samples were identified, including patients 18, 25, 30, 31, 33, 40, and 44. In contrast, t-SNE, a nonlinear technique, effectively visualizes high-dimensional data clusters by preserving local structures (Fig. 4E) and shows results similar to those of hierarchical clustering (Fig. 4B) (*16*, *41*, *42*). However, t-SNE incorrectly classified patient 49 as part of the thrombosis-negative group, resulting in a false-negative prediction. We identified that the thrombosis-positive group harbors a higher risk (score = 2), including patients 47, 48, 50, 51, 52, and 53. Nevertheless, the distance between the thrombosis-positive group with a higher risk (score = 2) and the thrombosis-positive group with a lower risk (score = 1), including patients 3, 17, and 36, is small, leading to three false-positive predictions. UMAP maintains both local and global structures and offers faster computational speeds (Fig. 5F) (*16*, *41*, *42*). The single thrombosis-positive group, which includes patients 47, 48, 50, 51, 52, and 53, demonstrates the effectiveness of combining holistic data structures with nonlinear strategies for predicting thrombosis, especially when using biomarker concentrations as indicative features.

Our research focused on predicting acute thrombosis. The four selected biomarkers were associated with acute responses (*5*, *43*). The pattern of these antigens being predominantly at low or high levels, rather than intermediate levels, was characteristic of acute response markers. These markers typically exhibited a binary expression pattern, reflecting a rapid clinical response to a triggering event, such as acute thrombosis formation. In the context of acute-phase responses, the immune system quickly up-regulated the production of these markers during an acute event, leading to a sharp increase in their levels. Conversely, in the absence of such an event, these markers remained at baseline, low levels. The absence of intermediate levels suggested that the regulatory mechanisms controlling these markers function in a switch-like manner, turning on or off rather than increasing or decreasing gradually (*5*, *43*).

In contrast, we used a “4-bit barcode” method (fig. S5) to evaluate thrombotic risk, compared to the “accurate four-biomarker concentration” method (Fig. 4). Low biomarker concentrations were coded as 0, while high concentrations were coded as 1. We applied the 4-bit barcode method as an input feature in various unsupervised machine learning models, including hierarchical clustering (fig. S5, B and C), PCA (fig. S5D), t-SNE (fig. S5E), and UMAP (fig. S5F). The performance of the 4-bit barcode method for acute thrombosis prediction was slightly worse than the accurate four-biomarker concentration method. Specifically, both the 4-bit barcode method (fig. S5, B and C) and the accurate four-biomarker concentration method (Fig. 4, B and C) produced a false negative for patient 49 in the hierarchical clustering analysis. In the thrombosis-negative group, the accurate four-biomarker concentration method performed better than the 4-bit barcode method. Only patients 3, 17, and 36 received false-positive predictions using the accurate four-biomarker concentration method in the hierarchical clustering analysis (Fig. 4, B and C). However, patients 3, 6, 17, 36, and 46 received false positives using the 4-bit barcode method in the hierarchical clustering analysis (fig. S5, B and C).

In addition, it was difficult to distinguish between the thrombosis-positive and thrombosis-negative groups in the PCA analysis using the 4-bit barcode method (fig. S5D). The t-SNE analysis using the 4-bit barcode method also incorrectly classified patient 49 as part of the thrombosis-negative group, resulting in a false-negative prediction, while identifying patients 3, 6, 17, 36, and 46 as part of the thrombosis-positive group, leading to false positives (fig. S5E). Similarly, the UMAP analysis using the 4-bit barcode method incorrectly classified patient 49 as part of the thrombosis-negative group, resulting in a false negative, and classified patients 3, 6, 12, 17, 36, and 46 as part of the thrombosis-positive group, leading to false positives (fig. S5F). Despite these discrepancies, the 4-bit barcode method (fig. S5) remains an effective strategy for acute thrombosis prediction and has a comparable, although slightly worse, predictive capability for acute thrombotic events in the COVID-19 cohort compared to the accurate four-biomarker concentration method (Fig. 4).

### Statistical analysis to evaluate different combinations of biomarkers

On the basis of the ICD-10 diagnostic code, patients 1 to 46 were negative for thrombosis, and patients 47 to 53 were positive. Specifically, we juxtaposed the two cohorts: those tested negative (−) and those positive (+) for thrombosis (Fig. 5, A to D). The various concentrations of CRP (Fig. 5A), calprotectin (Fig. 5B), sP-selectin (Fig. 5C), and D-dimer (Fig. 5D) were determined using the fabricated immunosensors. The accuracy of analyzing these four biomarkers was validated against ELISA as the reference standard (figs. S1 to S4). Of 53 specimens, CRP (*P* = 0.0191) and D-dimer (*P* = 0.0427) were statistically significant for predicting thrombosis (Fig. 5, A and D), whereas calprotectin (*P* = 0.0751) and sP-selectin (*P* = 0.1251) were not statistically significant (Fig. 5, B and C). In addition, the proximity matrix of four biomarkers and 53 patients was displayed in Fig. 5 (E and F), respectively.

A binary logistic regression analysis was performed, correlating the concentration of the four biomarkers of CRP, calprotectin, sP-selectin, and D-dimer with the ICD-10 code for thrombosis prediction. As delineated in Fig. 5 (G to U), an area under the receiver operating characteristic curve (AUC-ROC) value of 0.95 indicated a high true-positive rate (high sensitivity) and a low false-positive rate (1-specificity) when combining these four biomarkers for thrombosis prediction (Fig. 5K). Single-biomarker ROC values were lower than those of the combined biomarkers: CRP (AUC: 0.773) (Fig. 5G), calprotectin (AUC: 0.711) (Fig. 5H), sP-selectin (AUC: 0.683) (Fig. 5I), and D-dimer (AUC: 0.739) (Fig. 5J). In addition, we calculated the AUC for combinations of two and three biomarkers. These AUC values were below the combination of four biomarkers (AUC: 0.95; Fig. 5K): CRP and calprotectin (AUC: 0.891; Fig. 5L); CRP and sP-selectin (AUC: 0.885; Fig. 5M); CRP and D-dimer (AUC: 0.929; Fig. 5N); calprotectin and sP-selectin (AUC: 0.879; Fig. 5O); calprotectin and D-dimer (AUC: 0.919; Fig. 5P); sP-selectin and D-dimer (AUC: 0.916; Fig. 5Q); CRP, calprotectin, and sP-selectin (AUC: 0.885; Fig. 5R); CRP, calprotectin, and D-dimer (AUC: 0.916; Fig. 5S); CRP, sP-selectin, and D-dimer (AUC: 0.916; Fig. 5T); calprotectin, sP-selectin, and D-dimer (AUC: 0.929; Fig. 5U). Thus, combining the four biomarkers of CRP, calprotectin, sP-selectin, and D-dimer demonstrates high sensitivity and specificity for thrombosis prediction.

Specifically, using only CRP and D-dimer yielded an AUC of 0.929, which is close to the four-biomarker AUC of 0.95. Among the 53 specimens, CRP (*P* = 0.0191) and D-dimer (*P* = 0.0427) were statistically significant for predicting thrombosis (Fig. 5, A and D), while calprotectin (*P* = 0.0751) and sP-selectin (*P* = 0.1251) were not statistically significant (Fig. 5, B and C). Among the four biomarkers evaluated, CRP exhibited the most significant statistical association with thrombosis, as evidenced by the lowest *P* value (*P* = 0.0191) and the highest area under the curve (AUC = 0.773) in ROC analysis, outperforming calprotectin, sP-selectin, and D-dimer. CRP is an acute-phase protein produced by the liver in response to inflammatory cytokines, with levels markedly elevated in patients with COVID-19, correlating positively with disease severity and mortality (*44*, *45*). Retrospective studies have consistently demonstrated a positive correlation between CRP levels and COVID-19 severity, further establishing its role in predicting thrombosis risk (*44*, *45*). When incorporated into predictive models, CRP’s low detection limits enhance the sensitivity of the model, particularly in detecting early or subclinical inflammatory changes that may precede thrombotic events. This attribute positions CRP as an effective early warning signal within a layered prediction model, warranting closer monitoring or additional testing with more specific biomarkers. While CRP is highly sensitive, its lack of specificity for thrombosis necessitates its use within a multibiomarker framework to achieve a balance between sensitivity and specificity. The proposed four-biomarker combination ensures that the predictive model remains responsive to early inflammatory changes while maintaining precision in identifying thrombotic events.

## DISCUSSION

Our machine learning–assisted prediction, using nanoengineered immunosensors that detect multiple circulating biomarkers, represents an accurate and rapid strategy for predicting acute blood clots. We used fiber laser engraving and CO_2_ laser cutting techniques to create microchannels and used electrochemical deposition of Au NPs on CNTs for conjugation with aptamers and antibody. This approach enabled high-throughput fabrication of nanoengineered multichannel immunosensors. Our strategy provides rapid electrochemical detection of multiple biomarkers, followed by unsupervised clustering, to enhance thrombosis prediction.

The nanoengineered multichannel immunosensor was fabricated using fiber laser–engraved CNTs for microelectrodes and CO_2_ laser cutting for microfluidic channels, resulting in a large surface area for the electrochemical deposition of Au NPs and conjugation with specific aptamers and antibody. Electrical impedance techniques were highly effective in assessing the binding affinity at the recognition components of the aptamers and antibody, and the conductive surfaces altered the charge-transfer resistance (*R*_ct_) at the electrode interface (*30*). Electrochemical impedance spectra showed a robust linear relationship between various concentrations of the targeted biomarkers and *R*_ct_, as depicted in Fig. 2F. As the concentration of biomarkers increased, response signals showed a proportional rise. This linearity persisted with minimal variation during the dissociation phase. The interactions were attenuated in a pH 2 buffer solution when rejuvenating the immunosensor surface, suggesting the presence of strong noncovalent forces, including hydrogen bonding and Van der Waals interactions (*37*). The established LODs (S/N = 3) were 0.023 μg ml^−1^ for CRP, 0.035 ng ml^−1^ for calprotectin, 0.019 ng ml^−1^ for sP-selectin, and 0.035 ng ml^−1^ for D-dimer, respectively.

In response to the urgency to combat acute illness–induced thrombosis, we fabricated nanoengineered multichannel immunosensors designed to quantify specific circulating biomarkers. Now, ELISAs are the reference standard for protein detection and quantification (*20*). The LOD in the “sandwich” ELISA strategy is determined by the binding affinity and specificity of the selected antibody pairs, which were screened and purchased from Thermo Fisher Scientific for optimal performance (note S2 and fig. S7) (*46*, *47*). However, ELISAs are limited by several experimental constraints that affect the rapid and accurate prediction of thrombosis. These include multistep labeling processes, costly labeling reagents, and extended detection durations (*20*, *48*). In this context, the development of our nanoengineered multichannel immunosensor is unique for its rapid fabrication, specific targeting of biomarkers, and broad detection spectrum. This enables a customized solution for early prediction of acute illnesses, thus preventing thrombosis-associated complications in vital organ systems.

Blood clots from thrombosis are well-recognized to cause complications, including pulmonary embolism in the lungs, stroke in the brain, and acute coronary syndrome in the heart, all of which are associated with high morbidity and mortality (*5*, *49*). CRP is primarily synthesized in the liver in response to inflammatory cytokines, with interleukin-6 (IL-6) being an essential inducer. As an acute-phase protein, CRP levels increase rapidly during systemic inflammation, making it a crucial biomarker for assessing the inflammatory status of patients. Mounting evidence supports a positive correlation between CRP levels and the severity of COVID-19. In contrast to IL-6, CRP levels have emerged as a potential predictive marker for the risk of thrombosis in these patients (*5*, *50*). Calprotectin levels (S100A8/S100A9), a cytosolic component of neutrophils, have been independently associated with thrombosis (*5*, *51*). Upon activation, platelets express P-selectin, a molecule adept at binding to and activating leukocytes. The vascular inflammatory response induces endothelial cell dysfunction, accompanied by elevated levels of adhesive molecules like P-selectin, which promote thrombus formation (*5*, *50*). This process underscores the interconnected roles of inflammation and thrombosis, particularly in disease states like COVID-19, where systemic inflammation is prevalent. Subsequent enzymatic processes can cleave P-selectin into its soluble form. D-dimer, the fibrin degradation product, is a biomarker for coagulation and fibrinolysis. D-dimer levels may serve as a valuable tool for thrombosis screening (*5*). However, factors such as disease severity, progression, and medication use can vary between patients, limiting the interpretation of changes in individual biomarkers. For the selection of four specific biomarkers associated with acute thrombosis, we followed the consensus of the COVID International Thrombosis Biomarkers Colloquium, which recommended a panel of individual biomarkers to predict the risk of developing thrombosis (*5*). We then proposed our strategy of combining four biomarkers for acute thrombosis prediction. CRP and calprotectin are typically elevated in various inflammatory and acute infection states (*44*, *52*, *53*). In addition, we included sP-selectin and D-dimer, which are associated with thrombosis risk. Thus, we developed a strategy that combines these four biomarkers for acute thrombosis prediction. Using this approach, we accurately predicted thrombosis in 49 of 53 patient specimens through unsupervised clustering based on the concentrations of the four biomarkers (Fig. 4). In addition, we demonstrated that the combined four biomarkers could enhance the sensitivity and specificity for thrombosis prediction (Fig. 5).

In our cohort of 53 blood specimens collected from hospitalized patients with COVID-19, single biomarkers proved insufficient for predicting thrombotic risk due to inadequate sensitivity and specificity. However, combining all four biomarkers in an integrative approach greatly improved prediction (*5*, *13*, *14*). Four anomalies were identified within our blood specimens: Patient 49 rendered a false-negative outcome, and patients 3, 17, and 36 were misclassified during unsupervised hierarchical clustering. Despite this, the thrombotic risk prediction scores for the other 49 patients were consistent with their clinical evaluations. Notably, patient 49 was correctly classified within the thrombosis-positive group using the PCA algorithm, while patients 3, 17, and 36 were classified as thrombosis negative using the UMAP algorithm. This emphasizes the need for multiple unsupervised learning techniques and cross-comparisons.

In our study on acute thrombosis prediction, we demonstrated that combining four biomarkers enhanced the sensitivity and specificity of predicting acute thrombosis using 53 blood specimens from hospitalized patients with COVID-19 (Fig. 5). We successfully predicted 43 of 46 specimens in the thrombosis-negative group and 6 of 7 in the thrombosis-positive group, achieving an overall accuracy of 49 of 53 patient specimens (Fig. 4). Only COVID-19 patient samples were analyzed, with the majority showing no indication of thrombotic risk. This focus raises concerns about the generalizability of our findings to other patient populations with different underlying conditions and risk profiles. Specifically, patients with cancer, patients with HIV, patients with trauma and hemorrhage, patients with sepsis, and those on anticoagulants exhibit distinct pathophysiological mechanisms that may influence biomarker expression differently from patients with COVID-19. For instance, patients with cancer often present a hypercoagulable state due to tumor-associated procoagulant factors, while patients with HIV may experience chronic immune activation that affects coagulation pathways (*54*, *55*). Patients on anticoagulants, meanwhile, might exhibit suppressed biomarker levels, potentially leading to false negatives if not accounted for in the predictive model. This represents a limitation of our research.

If we extend our current nanoengineered multichannel immunosensor with unsupervised clustering to detect patients with cancer, patients with HIV, patients with trauma and hemorrhage, patients with sepsis, or those on anticoagulants, then the selected combination of four biomarkers specific to acute thrombosis (*5*) may not effectively predict these conditions based on the current biomarker concentrations. However, when analyzed through unsupervised clustering, our strategy using the nanoengineered multichannel immunosensor holds potential for predicting these diseases. The layer-by-layer procedures for manufacturing the nanoengineered multichannel immunosensor and the unsupervised clustering analysis strategy are detailed in Materials and Methods. If a panel of biomarkers associated with these diseases is identified, then the nanoengineered immunosensor can be tailored to detect these biomarkers by modifying the corresponding aptamers or antibodies on the sensing electrodes. These biomarker concentrations can then be used as inputs for disease prediction models using unsupervised machine learning, generating a disease risk score for conditions such as cancer, HIV, hemorrhagic trauma, sepsis, and for anticoagulated patients. On the basis of multiple biomarkers, this strategy is expected to provide an accurate and efficient method for predicting various diseases and guiding personalized medicine.

The sP-selectin is a marker of platelet activation and endothelial dysfunction, and its elevation can be associated with both arterial and venous thrombosis (*5*, *50*, *56*). However, it is more strongly linked to arterial thrombosis due to its association with platelet activation, as platelet-rich thrombi are commonly involved in myocardial infarction or ischemic stroke. While elevated sP-selectin is more indicative of arterial thrombosis, elevated D-dimer is nonspecific and can indicate the presence of a thrombus in either the arterial or venous system. Elevated levels of CRP and calprotectin, both markers of inflammation, are also linked to thrombosis, but neither definitively indicates arterial versus venous thrombosis. Given the stronger association of sP-selectin with arterial thrombosis, combining elevated sP-selectin with CRP, calprotectin, and D-dimer levels could help predict the location of arterial thrombosis. This information can guide anticoagulation strategies, such as choosing between antiplatelet or antithrombotic therapy (*57*, *58*).

Now, our detection strategy requires approximately 1.5 hours to provide a thrombotic risk score. This includes about 25 min for blood collection and processing, 1 hour for the simultaneous detection of four biomarkers using multichannel techniques, and around 5 min for unsupervised machine learning calculations to determine the thrombotic risk score. In comparison, the commercial ELISA takes about 5 hours to detect a single biomarker. While our proposed detection strategy shortens the detection time and demonstrates high accuracy, the acute thrombosis readouts highlight the need for bedside, point-of-care technology with a readout time of less than 15 min and minimal user interaction. In addition, the relatively slow generation of plasma could affect clinical decision-making for acute coronary syndromes, stroke, pulmonary embolism, or traumatic injury. With emerging automation techniques that integrate blood collection and processing, multichannel detection, and unsupervised machine learning into a single system, we anticipate a shortened turnaround time by minimizing manual operations and further reducing detection time (*59*, *60*).

The strengths and shortcomings of the current clinical assays [e.g., aPTT (Activated Partial Thromboplastin Time), PT (Prothrombin Time), near-infrared (NIR), and clotting time] and the proposed strategy [nanoengineered multichannel immunosensor with unsupervised clustering (NMIUC)] in this research are summarized in table S6. In summary, while aPTT and PT are effective for monitoring anticoagulation therapy, they lack specificity for predicting acute thrombosis. NIR shows promise for noninvasive monitoring but has not yet been widely adopted in clinical practice and may exhibit variable sensitivity and specificity. Clotting time is a quick test but is unreliable for thrombosis risk prediction due to its limited specificity and sensitivity. In contrast, our proposed strategy offers advantages, including high sensitivity, specificity, and accuracy for acute thrombosis prediction, while being simple, rapid, cost-effective, and customizable for multiple biomarkers. The proposed strategy is also readily accessible and user-friendly for blood testing, providing thrombotic risk assessments that could aid in acute thrombosis management and support personalized patient care. However, current limitations include validation restricted to only 53 specimens and its use being limited to research settings. Our NMIUC provides a fundamental and experimental basis to expand validation to larger cohorts for future clinical translation.

Furthermore, including diverse clinical data (e.g., sex, age, and thrombosis risk factors) and medical history (e.g., antiplatelet and anticoagulation drugs) is crucial for conducting a comprehensive analysis to strengthen this research (*5*, *61*, *62*). The diversity of clinical data enhances the generalizability of acute thrombosis prediction. Specifically, men and women often have different risk profiles for thrombosis due to biological differences such as hormone levels, genetic factors, and immune responses (*5*, *61*, *62*). Incorporating sex into the model helps tailor predictions to these differences and ensures that both sexes are adequately represented, improving accuracy across different patient populations. Thrombosis risk generally increases with age due to factors like reduced mobility, increased incidence of comorbidities, and changes in the coagulation system (*5*, *61*, *62*). Including age in the prediction model allows for consideration of these age-related variations, enabling risk stratification into different categories for personalized predictions. Incorporating a wide range of thrombosis risk factors allows the model to provide a more comprehensive risk assessment, capturing complex interactions between factors. Some risk factors may have a more substantial impact when combined, and accounting for these interactions improves the predictive power of the model. Patients on antiplatelet or anticoagulation therapy have modified thrombosis risk (*61*). While these medications are designed to reduce clot formation, their effectiveness can vary on the basis of individual patient factors. Including this information in the model helps account for the protective effects of these drugs and potential variations in their efficacy. In summary, the diversity of clinical data is essential for developing a robust acute thrombosis prediction model. By incorporating factors such as sex, age, thrombosis risk factors, and medication use, the model can better account for individual variability, resulting in more personalized and reliable predictions.

In summary, our study elucidates the combined utility of four biomarkers of CRP, calprotectin, sP-selectin, and D-dimer in augmenting the prediction of acute blood clots. This enhancement is achieved by applying nanoengineered multichannel immunosensors with unsupervised learning techniques for clustering and analysis. The nanoengineered multichannel immunosensor allows for sensitive and specific detection of a wide range of biomarker concentrations, facilitating unsupervised clustering. Thus, the nanoengineered multichannel immunosensor provides accurate and rapid prediction of acute blood blots, enabling timely responses to acute illnesses and public health crises.

## MATERIALS AND METHODS

### Materials and reagents

Gold (III) chloride hydrate (~52% Au basis), hexachloroplatinic (IV) acid hydrate (~40% Pt basis), silver (I) nitrate (≥99.0%), potassium chloride hydrate, sodium hypochlorite solution (available chlorine, 4.00 to 4.99%), sulfuric acid (95.0 to 98.0%), nitric acid (70%), potassium hexacyanoferrate (II) trihydrate (≥98.5%), potassium ferricyanide (III) (99%), toluene (99.9%), xylene (99%), Hepes solution, Tween 20, glycine (≥98.5%), silver chloride (99%), ethanol (≥99.5%), sodium chloride (≥99.0%), PBS [1.0 M (pH 7.4)], NHS (98%), EDC (≥97.0%), and human CRP, human calprotectin, human sP-selectin, human D-dimer, and bovine serum albumin were all purchased from Sigma-Aldrich.

The CNTs (XFM01, multiwalled, 5 to 15 nm in diameter, 10 to 30 μm in length) were sourced from XFNANO Technology Co. Ltd. The PVC substrate was procured commercially from Takiron Co. Ltd. Styrene–isoprene styrene (SIS; D1113) elastomer was acquired from Kraton Corporation. Adhesive Very High Bond (VHB) tapes were obtained from 3M Company. The aptamers were synthesized and purified by Integrated DNA Technologies (table S5). The anti-calprotectin (S100A8/S100A9, MABF291) antibody was purchased from Sigma-Aldrich. The Invitrogen CRP Human ELISA Kit, Invitrogen Calprotectin Human ELISA Kit, Invitrogen sP-selectin Human ELISA Kit, and Invitrogen D-Dimer Human ELISA Kit were all obtained from Thermo Fisher Scientific. The ultrapure water (18.2 megohm·cm) was purified using a Millipore system.

### Fabrication of a nanoengineered multichannel immunosensor

SIS (1 g) was initially dissolved in a 20-ml solvent mixture of toluene and xylene at a 1:1 ratio. This SIS solution was subsequently sprayed onto a PVC substrate (layer 1) to form an adhesive layer (layer 2). Acid-treated CNTs were ultrasonically dispersed in ethanol and sprayed onto the SIS adhesive layer (layer 2). Electrodes and connection wires for the CNTs (layer 3) were patterned using selective laser ablation via a 1064-nm Monport 30W fiber laser engraver and a marking system. The CNT connection wires were subsequently encapsulated using a diluted SIS solution applied through a predesigned mask, forming layer 4. For the preparation of layer 5, the Au NPs, Pt NPs, and Ag NPs were electrochemically deposited onto the four working electrodes, the counter electrode, and the reference electrode using gold (III) chloride hydrate, hexachloroplatinic (IV) acid hydrate, and silver (I) nitrate solutions, respectively. The Ag NPs were then chloridized using a diluted sodium hypochlorite solution, resulting in AgCl/Ag NPs.

For the sixth layer of the immunosensor, thiol-modified aptamers (50 μM) specific to CRP, sP-selectin, and D-dimer were incubated on the Au NP working electrodes for 1 hour, producing the aptamer/AuNP/CNT electrode. To modify the calprotectin antibody onto the carboxylated CNTs, the CNTs were initially incubated in a solution containing EDC (0.15 M) and NHS (0.1 M) for 1 hour to activate the carboxylic groups. This was followed by adding the calprotectin antibody solution and incubating for an hour.

The detection chamber (layer 7), the washing chamber cover (layer 8) with specific flow-directing holes, the chamber (layer 9), and the top cover (layer 10) were patterned using a 50-W CO_2_ laser cutter from Universal Laser Systems. A 3M VHB double-sided tape was patterned to incorporate four secondary chambers as the detection chamber (layer 7). Using the CO_2_ laser, four distinct holes were cut into a 0.1-mm-thick PVC film for the washing chamber cover (layer 8). The washing chamber (layer 9) featured a unified flow channel for waste liquid collection. Last, another PVC film was cut to introduce a hole as the liquid entrance for the top cover (layer 10).

### Material characterization

XPS measurements were conducted using a Kratos AXIS Ultra DLD spectrometer. High-resolution transmission electron microscopy images and EDX elemental mapping were acquired with an FEI TITAN transmission electron microscope operating at 120 kV.

### Operational workflow of the nanoengineered multichannel immunosensor

The priming of the chip, loading with plasma, signal acquisition, and regeneration of the nanoengineered multichannel immunosensor are shown in fig. S6 in the Supplementary Materials. Briefly, the immunosensor was washed with PBS (pH 7.4; 1 ml, repeated three times) before use. A 100-fold diluted plasma sample (0.2 ml) was pipetted into the immunosensor via the inlet. After a 45-min incubation to allow for the formation of noncovalent solid interactions between the targeted biomarkers in each of the four chambers and the modified aptamers or antibody, the residual plasma was removed with PBS (pH 7.4; 1 ml, repeated three times). Electrochemical measurements were conducted using a Gamry Interface 1010E workstation (Gamry Instruments Inc., USA). EIS was performed over a frequency range of 100 kHz to 0.1 Hz. The EIS data were fitted to the Randles model to extrapolate the *R*_ct_ parameter (*38*). CV was performed with potentials set between 0.4 and 1.3 V (versus the reversible hydrogen electrode) at a scan rate of 50 mV/s. For electrochemical measurements, the [Fe(CN)_6_]^3−/4−^ solution (0.2 ml) was pipetted into the individual detection chambers (measurement time: 5 min). After detecting the four biomarkers, the [Fe(CN)_6_]^3−/4−^ solution was removed from the detection chambers, which were then washed with PBS (pH 7.4; 1 ml, repeated three times). Subsequently, the immunosensor chambers were regenerated using 0.1 M glycine-HCl buffer (pH 2; 1 ml, repeated twice) to remove the bound biomarkers from the aptamers or antibody. Last, the chambers were washed with PBS and stored at 4°C for future use. We compared the performance of the fabricated nanoengineered multichannel immunosensor with previously reported immunosensors and demonstrated its high detection capabilities for the four biomarkers (tables S1 to S4) (*63*–*78*).

### Surface plasmon resonance

SPR measurements for binding kinetics were performed on a Biacore 2000 system. Assays were conducted at 25°C using a running buffer composed of 10 mM Hepes, 150 mM NaCl (pH 7.4), and 0.005% (v/v) Tween 20. The aptamer was immobilized onto a Series S Sensor Chip SA (GE Healthcare). A twofold dilution series of the specific biomarker was injected over the immobilized aptamer at a flow rate of 30 μl/min, with association and dissociation times of 180 and 900 s, respectively. The aptamer was regenerated using 0.1 M glycine-HCl buffer (pH 2) for two 10-s intervals.

### Ethics statement

All procedures and handling of human blood samples were conducted in accordance with the guidelines set by UCLA and received approval from the UCLA Institutional Review Board (no. IRB-23-1768). Diagnoses of SARS-CoV-2 infection and thrombosis were obtained from the UCLA Pathology Biobank and Biospecimen Research Core.

### Biosafety

All blood samples were processed in accordance with the biocontainment procedures established for handling SARS-CoV-2–positive samples. These specimens were approved by the UCLA Institutional Review Board protocol and provided by the UCLA Pathology Biobank and Biospecimen Research Core. All participants provided informed consent. The blood samples were deidentified, ensuring that no personally identifiable information was associated with them.

### Patient plasma samples

Patients testing positive for SARS-CoV-2 were recruited into the study subsequent to their diagnosis. The diagnosis of thrombosis was validated with the ICD-10 diagnostic code. Blood samples were handled in strict accordance with the biocontainment procedures designed for SARS-CoV-2–positive specimens. The details for blood collection and processing are provided in note S1 of the Supplementary Materials.

### Unsupervised hierarchical clustering and dimensionality reduction analysis

Hierarchical clustering and dimensionality reduction were executed using the Scikit-learn library in Python and GraphPad Prism 9 software. Furthermore, data visualization, including chord diagrams, was accomplished using Origin Lab 2021 and GraphPad Prism 9 software suites. Specifically, we used the Ward’s agglomerative hierarchical clustering technique, performing unsupervised clustering based on Euclidean distances, and emphasized optimizing the cluster count for the datasets. We used GraphPad Prism software for PCA and the Scikit-learn library in Python for nonlinear dimensionality reduction methods, including t-SNE and UMAP, to transform high-dimensional data into two-dimensional spaces.

### Statistical analysis

Statistical analyses were conducted using IBM SPSS Statistics 26, GraphPad Prism 9 software, and Python scripts. The *P* value was determined by the Mann-Whitney *U* test. A correlation was considered statistically significant at *P* values below 0.05. All data are presented as the mean ± SEM (*n* = 3), unless otherwise specified.

### ROC curves

To assess the precision of thrombosis prognosis, we tallied the numbers for true positives, true negatives, false positives, and false negatives. The ROC curve was illustrated on the basis of sensitivity and 1-specificity scores. The 95% confidence interval was computed for each AUC value. Binary logistic regression is a statistical method used to predict the probability of an event occurring given a set of predictor variables, when the outcome variable is binary: thrombosis (−) and thrombosis (+). A binary logistic regression model was initially calculated using the given dataset. Subsequently, the model’s predicted probabilities for the positive class were used to construct an ROC curve.

### Comparison with previously reported immunosensors and thrombosis prediction strategies

We compared the performance of the fabricated nanoengineered multichannel immunosensor with previously reported immunosensors (tables S1 to S4) (*63*–*78*). In addition, the strengths and shortcomings of current clinical assays (e.g., aPTT, PT, NIR, and clotting time) and the strategy proposed in this research are summarized in table S6 (*5*, *49*, *79*, *80*).

## Supplementary Materials

### This PDF file includes:

Supplementary Notes S1 and S2 Figs. S1 to S7 Tables S1 to S6
