# Supplementary material for: Rapid prediction of acute thrombosis via nanoengineered immunosensors with unsupervised clustering for multiple circulating biomarkers
Source: Sci Adv. 2024 Dec 11;10(50):eadq6778. doi: 10.1126/sciadv.adq6778 (PMC11633740; doi:10.1126/sciadv.adq6778)
Supplement: Supplementary file 1 — Supplementary Notes S1 and S2 Figs. S1 to S7 Tables S1 to S6 [file sciadv.adq6778_sm.pdf]

Science Advances
AAAS

Supplementary Materials for

Rapid prediction of acute thrombosis via nanoengineered immunosensors with unsupervised clustering for multiple circulating biomarkers

Kaidong Wang *et al.*

Corresponding author: Tzung K. Hsiai, thsiai@mednet.ucla.edu

Sci. Adv. **10**, eadq6778 (2024)
DOI: 10.1126/sciadv.adq6778

This PDF file includes:

Notes S1 and S2
Figs. S1 to S7
Tables S1 to S6

Note S1. Blood collection and processing

The first step involved collecting blood from the patient using standard phlebotomy techniques into tubes containing an anticoagulant to prevent clotting. The blood was then centrifuged at a low speed of 1500g for 10 minutes, separating it into three layers: red blood cells at the bottom, a buffy coat layer containing white blood cells and platelets in the middle, and plasma at the top. The top plasma layer was carefully aspirated using a pipette, taking care not to disturb the buffy coat layer, as this plasma may still contain some platelets. The aspirated plasma was then centrifuged at a higher speed of 3000g for 10 minutes, causing any remaining platelets to form a pellet at the bottom of the tube. After this second centrifugation, the supernatant (plasma) was carefully pipetted into a new sterile container, ensuring no disturbance of the platelet pellet. This supernatant was the prepared plasma used for testing thrombosis biomarkers with the multichannel nanoengineered immunosensor.

Note S2. The screening strategy for antibody pairs in the ELISA kit

For the "sandwich" ELISA strategy, the affinity of the antibody pair (two antibodies) is crucial in determining the limit of detection. During the screening process, potential antibody pairs were tested in various combinations of capture and detection antibodies to identify those with the highest affinity and specificity for the antigen. High-throughput screening methods, such as ELISA, were utilized to assess each antibody pair's binding affinity (the strength of binding to the antigen) and specificity (the ability to bind the antigen without cross-reacting with other molecules). Differences in the limits of detection for the targeted antigens could be attributed to variations in the binding affinity of the selected antibody pairs. The commercial company (Thermo Fisher Scientific, Cleveland, OH, USA, Invitrogen™) conducted extensive screening of antibody pairs using binding assays to select the high-affinity antibodies incorporated into their ELISA kits. We purchased these kits from Thermo Fisher Scientific (Cleveland, OH, USA, Invitrogen™) to acquire the necessary antibody pairs.

In our study, we employed a label-free strategy (one aptamer or antibody) for specific biomarker detection. The high-affinity aptamers targeting CRP, soluble P-selectin, and D-dimer were synthesized and purified by Integrated DNA Technologies (IDT). Aptamers, which can be synthesized in vitro via PCR methods, provide a cost-effective alternative to antibodies. Additionally, we purchased the antibody for calprotectin detection from Thermo Fisher Scientific (Cleveland, OH, USA). Our objective was to achieve rapid prediction of acute thrombosis. Therefore, we utilized a label-free detection strategy to reduce the detection time from ~ 5 hours to ~ 1.5 hour. This approach involved measuring electrochemical impedance signals generated by the formation of specific immune complexes, which act as a blocking layer and inhibit charge transfer on the surface of the sensing electrode. The Randles equivalent circuit was then used to calculate charge-transfer resistance (R_{ct}), which correlates with the biomarker concentration.

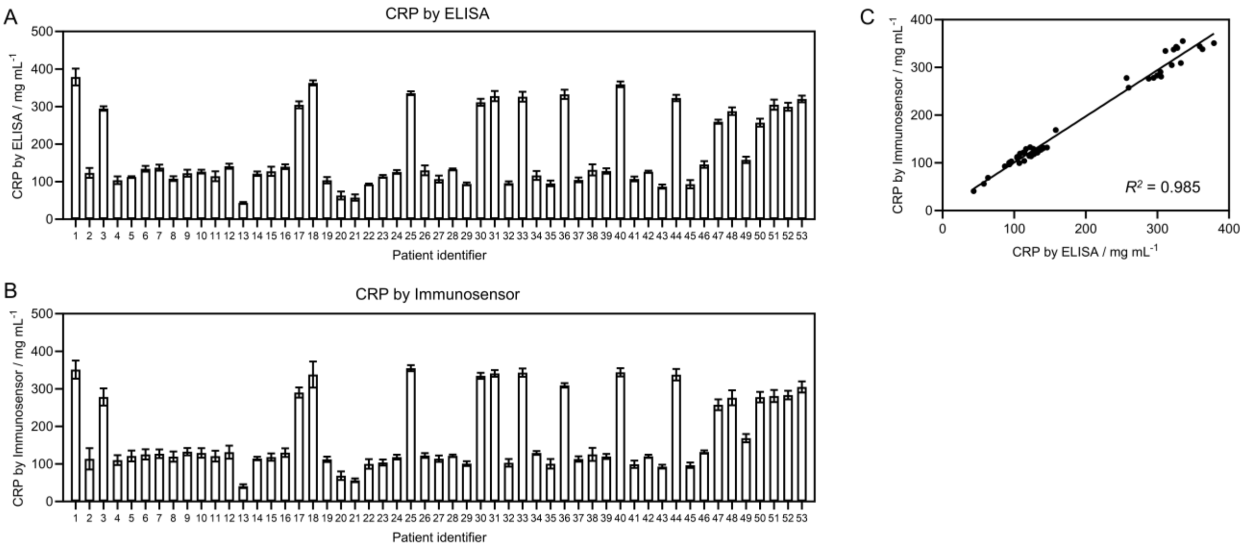

Fig. S1. The detection of CRP in human plasma samples was compared using the fabricated immunosensor and ELISA. (**A**) CRP detection utilizing the fabricated immunosensor, based on three independent experiments (n = 3). (**B**) CRP detection via ELISA, based on three independent experiments (n = 3). (**C**) Illustration of the linear relationship between results obtained from the fabricated immunosensor and those from ELISA. The Pearson correlation coefficient (R) was determined using linear regression analysis.

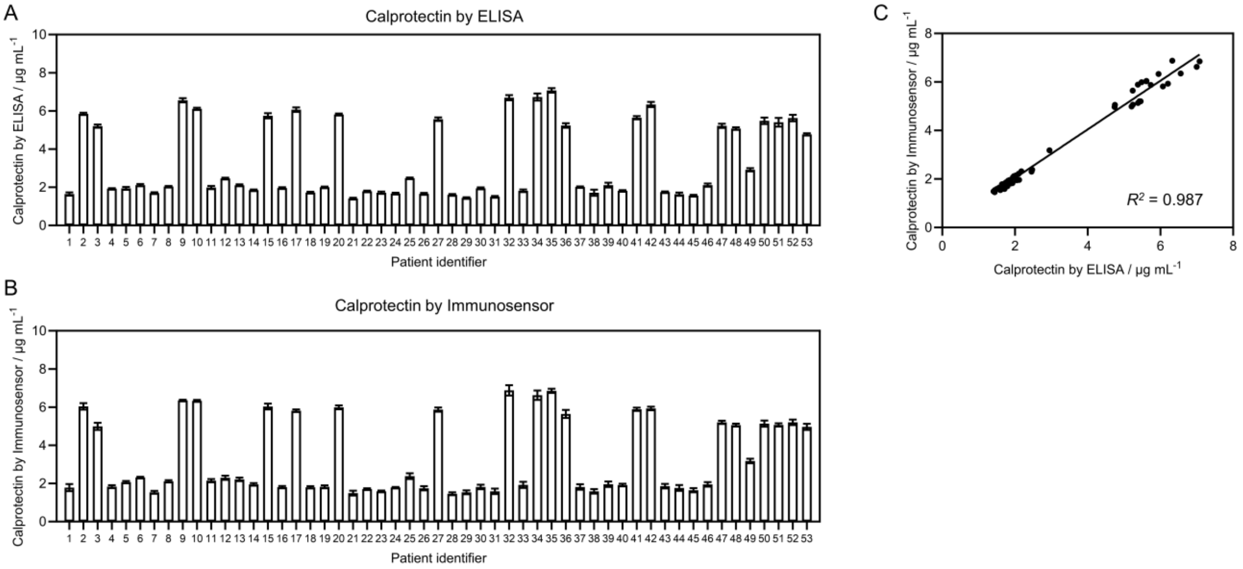

Fig. S2. The detection of calprotectin in human plasma samples was compared using the fabricated immunosensor and ELISA. (**A**) Calprotectin detection utilizing the fabricated immunosensor, based on three independent experiments (n = 3). (**B**) Calprotectin detection via ELISA, based on three independent experiments (n = 3). (**C**) Illustration of the linear relationship between results obtained from the fabricated immunosensor and those from ELISA. The Pearson correlation coefficient (R) was determined using linear regression analysis.

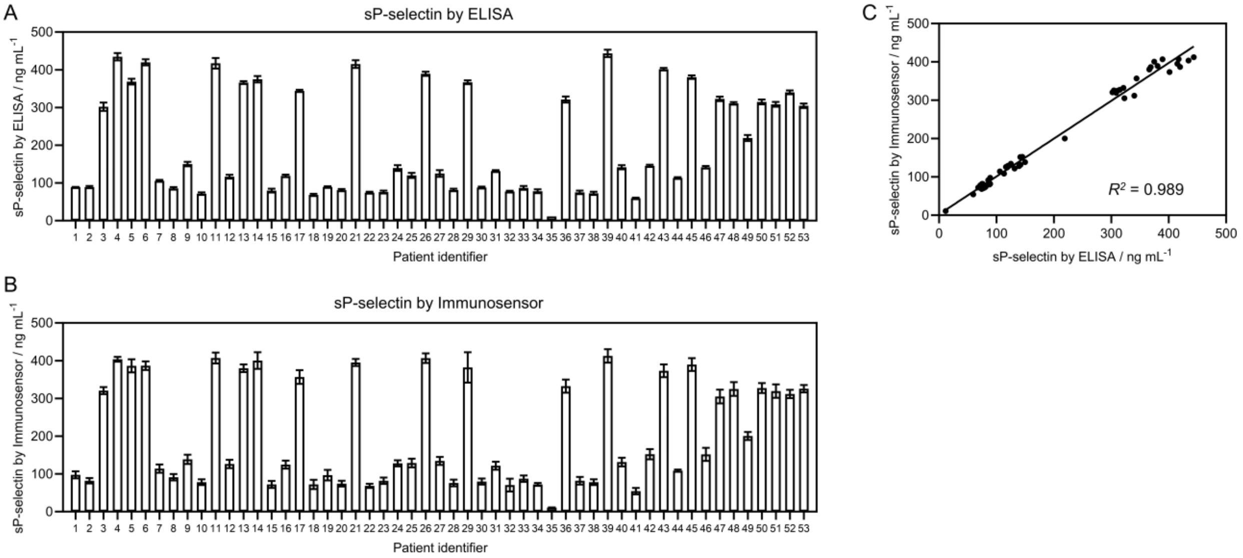

Fig. S3. The detection of sP-selectin in human plasma samples was compared using the fabricated immunosensor and ELISA. (**A**) sP-selectin detection utilizing the fabricated immunosensor, based on three independent experiments (n = 3). (**B**) sP-selectin detection via ELISA, based on three independent experiments (n = 3). (**C**) Illustration of the linear relationship between results obtained from the fabricated immunosensor and those from ELISA. The Pearson correlation coefficient (R) was determined using linear regression analysis.

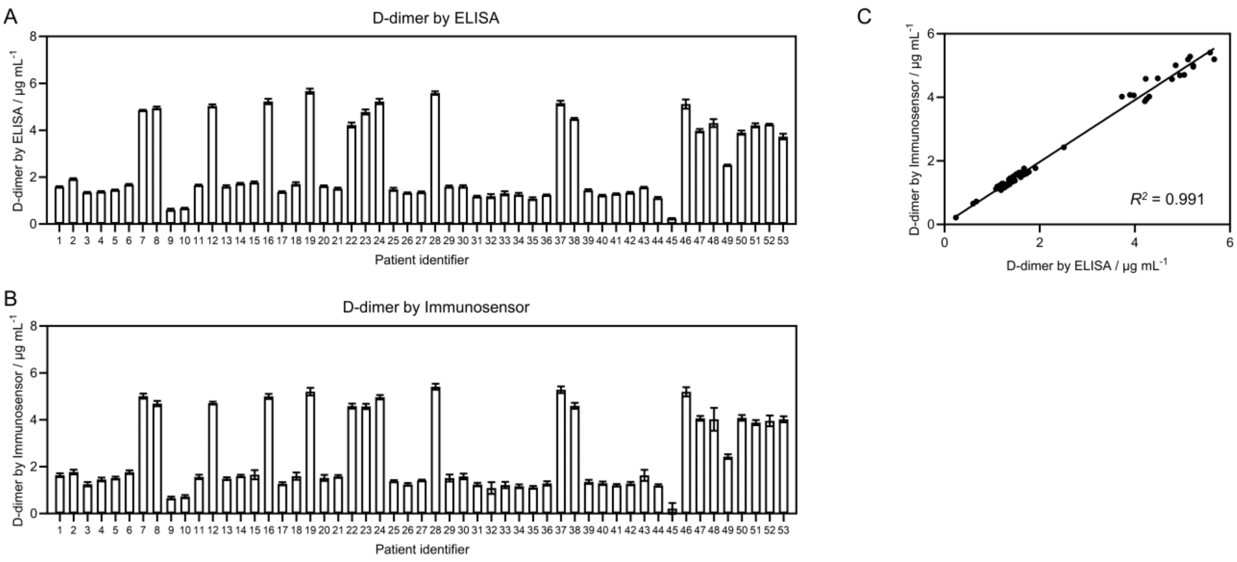

Fig. S4. The detection of D-dimer in human plasma samples was compared using the fabricated immunosensor and ELISA. (**A**) D-dimer detection utilizing the fabricated immunosensor, based on three independent experiments (*n* = 3). (**B**) D-dimer detection via ELISA, based on three independent experiments (*n* = 3). (**C**) Illustration of the linear relationship between results obtained from the fabricated immunosensor and those from ELISA. The Pearson correlation coefficient (*R*) was determined using linear regression analysis.

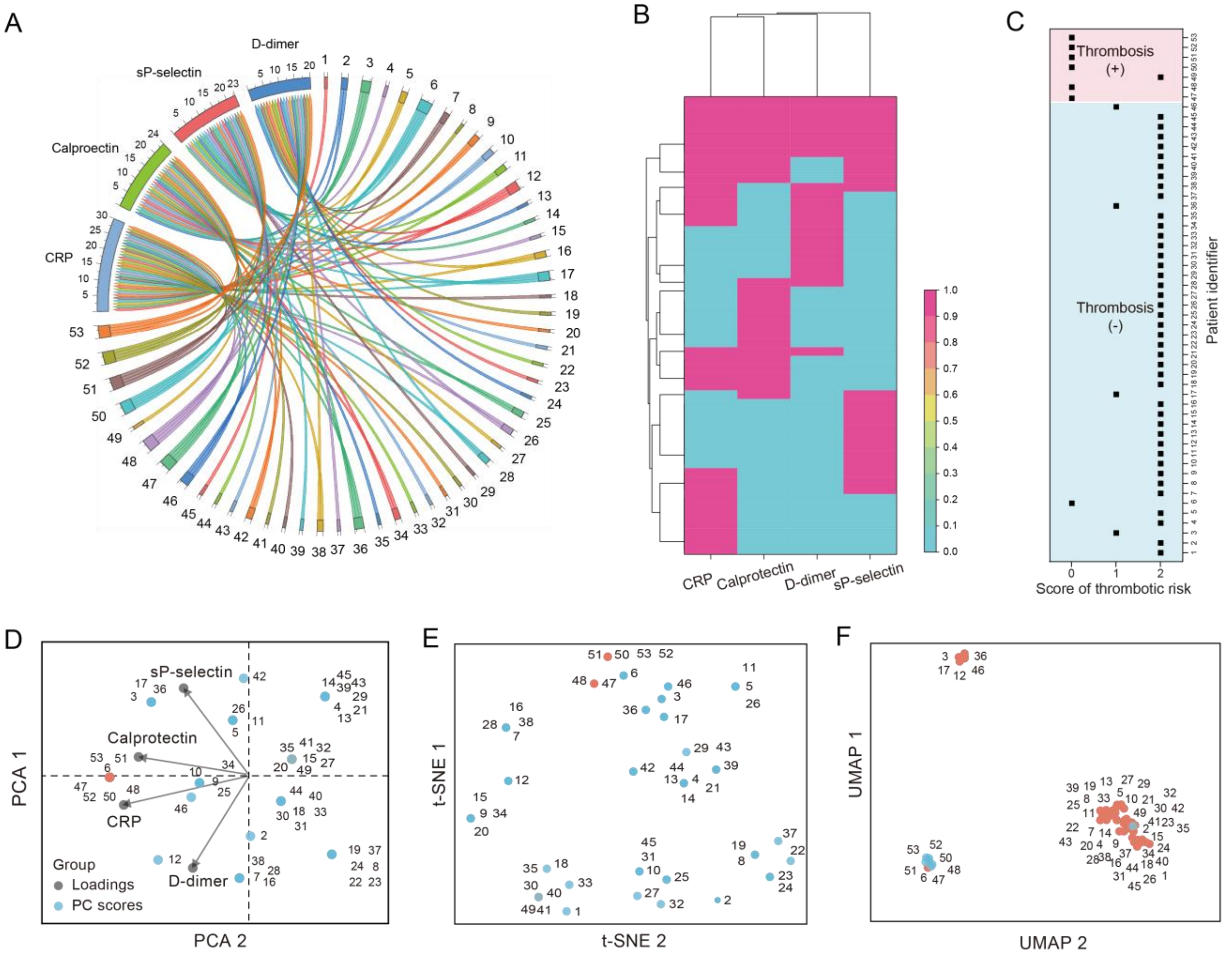

Fig. S5. Unsupervised clustering was performed using the "4-bit barcode" method for acute thrombosis prediction. (**A**) A chord diagram illustrating the network relationship among the 4 biomarkers and 53 patients. (**B**) Hierarchical clustering analysis for acute thrombosis prediction. (**C**) Hierarchical clustering outcomes validated against the ICD-10 diagnostic code for thrombosis. (**D**) PCA analysis for acute thrombosis prediction, where each arrow represents the influence of a different biomarker concentration in the PCA space. (**E**) t-SNE analysis for predicting acute thrombosis. (**F**) UMAP analysis for predicting acute thrombosis.

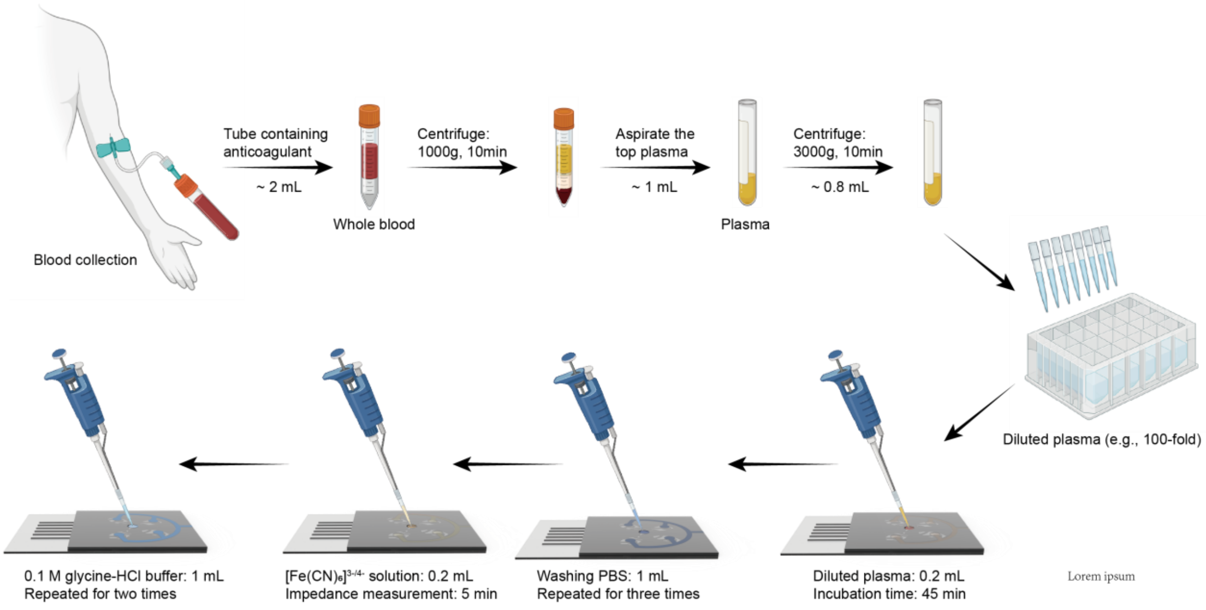

Fig. S6. A process diagram showed the steps for priming the chip, loading it with plasma, obtaining the signal, and regenerating the chip. The immunosensor was prepared by washing with PBS, loading a diluted plasma sample for 45 minutes of incubation to allow biomarker binding, followed by electrochemical measurements using $[Fe(CN)_6]^{3-/4-}$ solution and EIS, after which the chambers were washed, regenerated with glycine-HCl buffer, and stored at 4°C for future use.

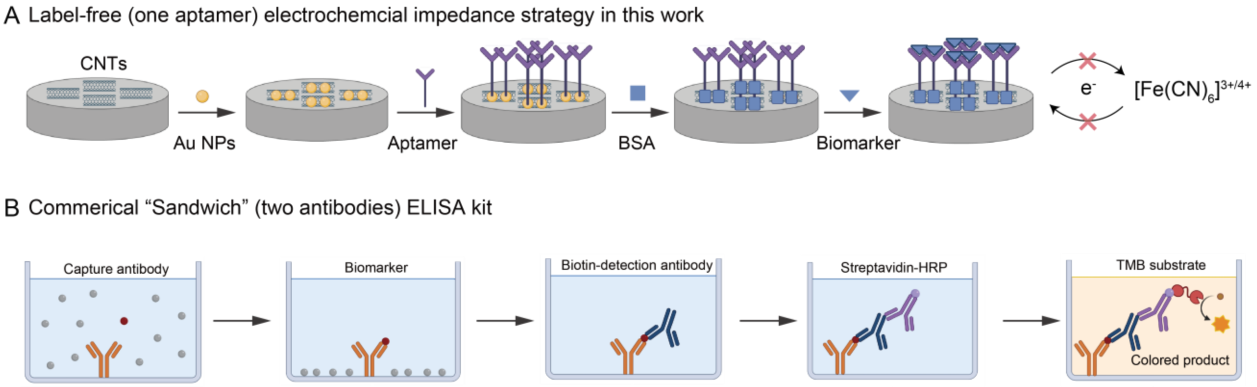

Fig. S7. A comparison of the label-free electrical impedance strategy and the "Sandwich" ELISA strategy was presented. (**A**) Principle of a label-free (one aptamer) electrochemical impedance strategy in this work. (**B**) Principle of commercial "Sandwich" (two antibodies) ELISA kit.

Table S1. Comparison of various immunosensors for CRP detection.

Strategy	Recognition Element	Detection Method	Detection Range ($\mu g\ mL^{-1}$)	Detection Limit ($\mu g\ mL^{-1}$)	Ref.
MSD	Antibody	Electroluminescent	$8\times10^{-6}-1$	8×10^{-6}	*(63)*
MNPs	Antibody	Labeled	$1.2-3.1\times10^{2}$	0.12	*(64)*
GNRs	Antibody	Labeled	$1\times10^{-3}–5\times10$	0.5×10^{-3}	*(65)*
Au	Aptamer	Label-free	$1.15\times10^{-1}-1.15\times10$	1.15×10^{-1}	*(66)*
CNTs	Aptamer	Label-free	$1\times10^{-1}-10^{4}$	0.023	This work

MSD: Multiplexed ELISA with mesoscale discovery reader. MNPs: Magnetic nanoparticles; GNRs: Gold nanorods; CNTs: Carbon nanotubes

Table S2. Comparison of various immunosensors for calprotectin detection.

Material	Recognition Element	Detection Strategy	Detection Range (ng mL^{-1})	Detection Limit (ng mL^{-1})	Reference
PtNi@CuMOFs	Antibody	Labeled	2×10^{-4}–5×10	1.377×10^{-4}	*(67)*
PS NPs	Antidboy	Labeled	3×10^{5}–2.47×10^{7}	3×10^{5}	*(68)*
PMMA sphere	Imprinted	Photonic	0.1–9.8	-	*(69)*
Ag@ZnO	Antibody	Labeled	1×10^{2}–1×10^{4}	1×10^{2}	*(70)*
CNTs	Antdiboy	Label-free	1×10^{-1}–10^{4}	0.035	This work

PtNi@CuMOFs: PtNi nanoparticles functionalized 2D Cu-metal organic framework nanosheets; PS NPs: polystyrene particles; PMMA: poly (methyl methacrylate); CNTs: Carbon nanotubes

Table S3. Comparison of various immunosensors for sP-selectin detection.

Material	Recognition Element	Detection Strategy	Detection Range (ng mL^{-1})	Detection Limit (ng mL^{-1})	Reference
-	Antibody	Labeled	1×10^{-2}–10	1×10^{-3}	*(71)*
CNTs@GNB	Antibody	Labeled	1×10^{-4}–1×10^{4}	8.5×10^{-4}	*(72)*
Peptidic β-sheet	Antibody	Labeled	1.1–3.5×10	1.1	*(73)*
-	Antibody	Labeled	0.5-1	0.5	*(74)*
CNTs	Aptamer	Label-free	1×10^{-1}–10^{4}	0.019	This work

CNTs: Carbon nanotubes; GNB: Gold nanobone

Table S4. Comparison of various immunosensors for D-dimer detection.

Material	Recognition Element	Detection Strategy	Detection Range (ng mL^{-1})	Detection Limit (ng mL^{-1})	Reference
Graphene	Antibody	Label-free	$1-10^3$.	3×10^{-1}	*(75)*
ZrO2	Antibody	Labeled	5×10^{-2}–6×10^2	2.1×10^{-2}	*(76)*
AuNCs	Antibody	Labeled	5×10^{-5}–1×10^2	2.92×10^{-5}	*(77)*
Polypyrrole	Antibody	Label-free	1×10^{-1}–5×10^2	1×10^{-1}	*(78)*
CNTs	Aptamer	Label-free	$1\times10^{-1}-10^4$	0.035	This work

AuNCs: Gold nanoclusters; CNTs: Carbon nanotubes

Table S5. The sequences of the aptamers.

Aptamers	Sequences
CRP	5’-GCCTGTAAGGTGGTCGGTGTGG<br>CGAGTGTGTTAGGAGAGATTGC-3’
sP-selectin	5’-ACGCUCAACGAGCCAGGAACAUCGACGU<br>CAGCAAACGCGAGCGCAACCAGUAACACC-3’
D-dimer	5’-GCGCGGTCCCGATTTGGTGT<br>AAAATTCCCTCAGCCCTACA-3’

Table S6. Comparison of the strengths and shortcomings of current clinical assays and the proposed strategy for predicting acute thrombosis.

Assay	Advantages	Disadvantages	Ref
aPTT	- Widely used and standardized.<br>- Useful for heparin therapy.<br>- Identifies abnormalities in coagulation pathways.	- Not specific for thrombosis.<br>- affected by various factors.<br>- Poor sensitivity to thrombosis risk	*(5)*
PT	- Widely available.<br>- Monitors warfarin therapy.<br>- Assesses extrinsic pathway function.	- Not specific for thrombosis.<br>- Affected by liver function.<br>- Limited predictive value for thrombosis risk.	*(49)*
NIR	- Non-invasive and can be used for real-time monitoring.<br>- Potential for assessing blood flow and tissue oxygenation.<br>- Capable of detecting changes blood characteristics.	- Requires specialized equipment and expertise.<br>- Limited to research settings, not widely adopted in clinics.<br>- Sensitivity and specificity are variable.	*(79)*
Clotting Time	- Simple and rapid test.<br>- Can provide a quick assessment of hemostasis.	- Influenced by various factors.<br>- Lacks specificity and sensitivity.	*(80)*
NMIUC	- Simple and rapid test.<br>- High sensitivity, specificity, and accuracy for acute thrombosis.<br>- Cost effectiveness and customizable for multiple biomarkers.	- Validation in 53 specimens.<br>- Limited to research settings, not widely adopted in clinics.	This work

aPTT: Activated partial thromboplastin time; PT: Prothrombin time; NIR: Near-infrared spectroscopy; MNIUC: Nanoengineered multichannel immunosensor with unsupervised clustering
